# Commentary: “Brain-Doping,” Is It a Real Threat?

**DOI:** 10.3389/fphys.2019.01489

**Published:** 2019-12-05

**Authors:** Zhiqiang Zhu, Junhong Zhou, Brad Manor, Xi Wang, Weijie Fu, Yu Liu

**Affiliations:** ^1^School of Kinesiology, Shanghai University of Sport, Shanghai, China; ^2^School of Physical Education, East China Jiao Tong University, Nanchang, China; ^3^Hinda and Arthur Marcus Institute for Aging Research, Hebrew SeniorLife, Boston, MA, United States; ^4^Harvard Medical School, Boston, MA, United States

**Keywords:** tDCS, sport/exercise performance, methodological hurdles, neuro-physiological mechanism, inter-personal differences, brain structure and function

## Introduction

On the basis of several recent meta-analyses synthesizing the effects of transcranial direct current stimulation (tDCS) on sport performance (Lattari et al., 2018; Machado et al., 2018; Holgado et al., 2019a), Holgado et al. (2019b) concluded that there is insufficient evidence to support an ergogenic or “brain-doping” effect of tDCS on sport and/or physical performance. The authors also highlighted that the exact neuro-modulatory mechanisms through which tDCS may improve human performance remain largely unclear. We describe here more explicitly several important limitations of the majority of tDCS research to date. We also examine potential mechanisms of action, and provide recommendations that we believe are needed to more robustly study the ergogenic effects of tDCS moving forward.

## Overcoming Methodological “Hurdles” in tDCS Research

Inconsistencies in tDCS effectiveness both within and between studies has been well-documented (Lattari et al., 2018; Machado et al., 2018; Holgado et al., 2019b) and likely arises from the combination of multiple issues. Across studies, numerous different devices have been utilized to administer stimulation, which may vary considerably in the current flow properties that they produce (Hahn et al., 2013; Zhang and Li, 2015). The characteristics of tDCS intervention, including the cortical target of interest, electrode size, electrode materials, and the intensity of current flow, have also varied considerably between studies, and moreover, are often insufficiently reported (Palm et al., 2014; Kouzani et al., 2016; Machado et al., 2018; Holgado et al., 2019a). Complicating matters further is that across subjects within studies, the same tDCS intervention may produce very different electrical fields in the cortex, and have different effects on brain function, due to inter-subject variation in both anatomy and physiology (Wiethoff et al., 2014; Li et al., 2015; Sanchez-Kuhn et al., 2018). Together, these challenges highlight the need for standardized reporting of tDCS, as well as the application of advanced technology to help measure and even “personalize” current flow.

Fortunately, recent technological advances offer promise to help researchers estimate the electric fields induced by tDCS, customize montages to individual head and brain anatomy, and examine the effects of tDCS on brain physiology. For example, Laakso et al. (2016) used a finite element modeling technique on a standardized brain template to determine the characteristics of the electrical field generated by tDCS. Such a technique now offers a data driven approach to developing tDCS montages that more likely target the cortical region of interest. Applying such a technique to individual brain MRIs also promises to help establish dose-response relationships between tDCS-induced electric fields and potential changes in functional performance. Beyond this, we believe that researchers should consider administering tDCS via an array of smaller electrodes, and in appropriate circumstances using montages created via modeling-based optimization techniques (Ruffini et al., 2014), to create more focal and “personalized” targets of tDCS (Madhavan and Stinear, 2010; Bikson et al., 2012; Li et al., 2015; Opitz et al., 2015). Together, we believe that the combination of these new techniques will ultimately help to produce larger, more consistent effects of tDCS intervention.

## Sport Performance is More than Strength and Endurance

Several published meta-analyses have combined data from studies on the effects of tDCS on both muscle strength and endurance (Machado et al., 2018; Holgado et al., 2019a). These two muscle functions stem from distinct neuro-physiologic procedures, are likely to be differentially affected by cortical neuromodulation, and should be considered separately in such analyses. Furthermore, numerous factors beyond muscle strength and endurance contribute to sports performance (Miller and Clapp, 2011; Loprinzi et al., 2013). Such factors as sensory perception and processing, fatigue, perceived exertion, multiple aspects of executive function including dual tasking and response inhibition, and neural recovery depend upon supraspinal function and appear to be modifiable via tDCS (Zhou et al., 2014; Wang et al., 2015; Vecchio et al., 2018; Angius et al., 2019) A better understanding of the acute and longer-term impact of tDCS on these factors is needed before definitive neuro-doping claims should be made.

## One Potential Mechanism of Action

Most studies to date have focused on the effects of tDCS on functional performance; e.g., tDCS targeting the bilateral motor cortex has been shown to improved cycling performance in healthy adults (Angius et al., 2018). Much more work is thus needed to uncover the neurophysiological mechanisms through which tDCS may improve such performance. Understanding this, in turn, will enable the development of neuromodulatory interventions directly aimed at enhancing such mechanisms.

Several potential mechanisms of action through which tDCS alters functional performance have been described. Such efforts have focused on the effects of tDCS on neurochemical transmitters (e.g., GABA, dopamine, adenosine) (Kuo et al., 2008; McLaren et al., 2018), as well as neurophysiologic responses (Labruna et al., 2019). GABA, for example, is one of the most important inhibitory neurotransmitters in the brain and has been linked to motor performance (Krause et al., 2013; Kolasinski et al., 2019). To this end, Kim et al. (2014) demonstrated that compared to cathodal or sham stimulation, anodal tDCS targeting the hand area of the left primary motor cortex induced a reduction in GABA concentration within this brain region. Excitingly, those who experienced a greater decrease in GABA concentration exhibited greater improvements in performance on both motor learning and motor memory tasks.

Additional work is also needed to better understand the links between tDCS-induced changes in neurophysiology and function outcomes. Labruna et al. (2019) recently published a promising study, for example, that used single-pulse transcranial magnetic stimulation (TMS) over the primary motor cortex to measure the effects of tDCS on resting motor thresholds (rMT). Results suggested that following tDCS, participants who exhibited a greater reduction in rMT tended to improve their performance more in a motor learning task. Moving forward, more studies such as these that combine both functional and mechanistic outcomes will help us better understand and optimize the effects of tDCS on sport performance.

## Is tDCS a “Neuro-Doping” Threat?

According to the current World Anti-Doping Code (WADA, 2019), a substance or method should be considered as “doping” if it meets two of three criteria based upon scientific criteria and/or experience: (1) it has potential beneficial effects on athletic performance, (2) it poses potential health risks to athletes, and (3) it violates the spirit of sport. We believe that tDCS has strong potential to enhance athletic performance, especially as advances in technology, modeling, and methodology help overcome the many challenges of traditional tDCS research. At the same time, tDCS is widely believed to pose non-significant risk to participants when recommended procedures are followed (Brunoni et al., 2011; Bikson et al., 2016; Jackson et al., 2017). tDCS therefore holds strong promise to improve performance without posing significant health risks to athletes. As such, we believe that determination of tDCS as a neuro-doping strategy will ultimately come down to the challenging ethical question of whether or not it negatively impacts the spirit of sport and fair competition.

## Author Contributions

ZZ, JZ, BM, XW, WF, and YL conceived, drafted, and revised the manuscript. All authors read and approved the final manuscript.

### Conflict of Interest

The authors declare that the research was conducted in the absence of any commercial or financial relationships that could be construed as a potential conflict of interest.
